# Multilevel Hollow‐Structured Particles through Halogen‐Bond Regulated Polymer Assembly under 3D Confinement

**DOI:** 10.1002/advs.202405103

**Published:** 2024-09-04

**Authors:** Xihuang Zheng, Yi Zhao, Yuping Zhang, Renhua Deng, Baohui Li, Senbin Chen, Jintao Zhu

**Affiliations:** ^1^ School of Chemistry and Chemical Engineering Huazhong University of Science and Technology (HUST) Wuhan 430074 China; ^2^ Key Laboratory of Weak‐Light Nonlinear Photonics, Ministry of Education, School of Physics Nankai University Tianjin 300071 China

**Keywords:** 3D confined assembly, amphiphilic triblock copolymer, halogen bonding interactions, selective swelling/deswelling

## Abstract

Engineering of hollow particles with tunable internal structures often requires complicated processes and/or invasive cleavage. Halogen‐bond driven 3D confined‐assembly of block copolymers has shed light on the engineering of polymer organization along with the fabricating of unique nanostructures. Herein, a family of multilevel hollow‐structured particles (e.g., fully porous, multi‐chamber, multi‐shell, and concentric multi‐layer architectures) is reported via halogen‐bond regulated 3D confined‐assembly of amphiphilic polymer networks. To do so, polystyrene‐*b*‐poly(2‐vinyl pyridine)‐*b*‐poly(ethylene oxide) (PS‐*b*‐P2VP‐*b*‐PEO) amphiphilic triblock copolymer is selected, where P2VP blocks act as halogen acceptor. Meanwhile, poly(3‐(2,3,5,6‐tetrafluoro‐4‐iodophenoxy) propyl acrylate) (PTFIPA) is employed as halogen donor. Halogen‐bond driven donor‐acceptor linking between PTFIPA and P2VP block presented in PS‐*b*‐P2VP‐*b*‐PEO, can lead to the formation of supramolecular polymeric networks, along with the increased P2VP domain and tunable hydrophobic volume. Therefore, an adjustable packing parameter (*p*) is thus anticipated, which can enable the morphology transformation sequence until an equilibrium state is reached. Moreover, computer simulations are further utilized as the tool to interpret such morphologies transition and identify the precise distribution of each component. Benefiting from the tunable hollow structure and a substantial surface for transporting purpose, these structurally novel particles open perspectives toward promising applications including encapsulation, nanoreactor, and catalyst support.

## Graphical abstract

Based on the studies combining experiments and simulations, halogen‐bond driven 3D confined‐assembly toward multi‐level hollow particles with tunable shape and internal structure (e.g., fully porous, multi‐chamber, multi‐shell, and concentric multi‐layer architectures) are systematically engineered. Polymer packing parameters and Monte Carlo simulations provide a reliable way to understand the dependence of morphology transition sequence.

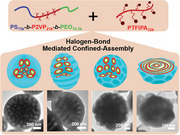

## 1 Introduction

Supramolecular forces driven macromolecular self‐assembly into nanostructured particles is of fundamental interest,^[^ ^1^, ^2^, ^3^, ^4^ ^]^ while a vital point in the exploring of these particles toward functional materials is mainly attributed to their tunable internal structures. Non‐covalent interactions (e.g., hydrogen‐bond,^[^ ^5^ ^]^ halogen‐bond,^[^ ^6^, ^7^, ^8^ ^]^ host–guest interaction,^[^ ^9^ ^]^ and metal‐ligand coordination,^[^ ^10^ ^]^ etc.) in tuning of dimensions.^[^ ^11^ ^]^ and types of internal nanostructures are considered to be essential, providing the specificity and responsivity required in many significant chemicals,^[^ ^12^ ^]^ biological.^[^ ^13^ ^]^ and medical.^[^ ^14^, ^15^, ^16^, ^17^, ^18^ ^]^ processes.

In virtue of the similarities and beneficial distinctions comparing to ubiquitous hydrogen‐bond (such as hydrophobicity, higher directionality, and being favored in polar solvents), halogen‐bond.^[^ ^6^, ^7^, ^19^ ^]^ has emerged as an engaging supramolecular tool toward molecular recognition. Encouraging examples have recently paved its way to a broad range of scientific endeavors, from anion sensing,^[^ ^20^, ^21^ ^]^ crystal engineering,^[^ ^22^ ^]^ organo‐catalysis,^[^ ^23^ ^]^ to the research domains of polymer/particle self‐assembly.^[^ ^24^, ^25^, ^26^, ^27^, ^28^ ^]^ and functional soft materials.^[^ ^8^, ^29^, ^30^, ^31^ ^]^

Although promising nanostructures can be anticipated from the engineering of halogen‐bonded polymers, few elegant studies of such attractive scaffolds have been reported so far.^[^ ^24^, ^25^, ^32^ ^]^ Moreover, aside from the nanostructures created via the solution or bulk self‐assembly, halogen‐bond regulated macromolecular confined assembly to promote the morphologies that must not be underestimated. Our interest is drawn by the possibility of utilizing halogen‐bond regulated macromolecules to promote the morphologies within emulsion droplets. Advantages of halogen‐bonded interpolymer complexation within emulsion droplets include the versatility and its potential to achieve novel dynamic and stimulus‐responsive structures.

PVP‐containing (e.g., P4VP‐ and P2VP‐) block copolymers (BCPs) identify themselves as significant functional blocks for generating smart multicompartment particles,^[^ ^33^, ^34^ ^]^ where the nitrogen center of the pyridyl motif presents a lone electron pair which do not overlap with aromatic heterocycles. Consequently, PVP‐containing BCPs are characterized by the basicity and association capability, respectively yielding pH‐responsive behavior and non‐covalently association, including halogen‐bond,^[^ ^24^ ^]^ hydrogen‐bond,^[^ ^35^ ^]^ and metal‐coordination interaction. We have recently reported an order‐to‐order morphology transition sequence within microparticles, for example, spherical → cylindrical → lamellar → inverse cylindrical, via halogen‐bond regulated 3D confined assembly of blending PS‐*b*‐P4VP with homopolymer PTFIPA (poly(3‐(2,3,5,6‐tetrafluoro‐4‐iodophenoxy)propyl acrylate).^[^ ^36^ ^]^

**Figure 1:**
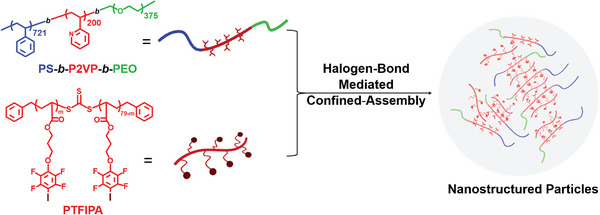
Schematic illustration of nanostructured particles, formed via halogen‐bond mediated 3D confined‐assembly between PS*‐b*‐P2VP‐*b*‐PEO amphiphilic triblock copolymer and PTFIPA homopolymer.

Differing from the abovementioned solid microspheres through halogen‐bonded hydrophobic macromolecules,^[^ ^36^ ^]^ one of the main new features investigated in this work is the use of PS‐*b*‐P2VP‐*b*‐PEO amphiphilic triblock copolymer. Therefore, upon mixing with PTFIPA homopolymer, halogen‐bond driven association between P2VP block and PTFIPA homopolymer facilitates the formation of an amphiphilic supramolecular polymer network. Moreover, the unique chain arrangement of the obtained amphiphilic supramolecular polymer networks within emulsion droplets is anticipated (**Figure** **1**). As the removal of organic solvent from oil‐in‐water emulsions will give rise to instabilities of oil/water interface, thus the creation of microparticles with novel hierarchical structures can be expected from such multicompartment co‐assembly. In addition, the beauty of halogen‐bond driven amphiphilic supramolecular networks lies in the tunable chain density by adjusting the PTFIPA loading amount, thus giving rise to the variation of building block ratios and polymer packing parameter, which could lead to the sequential nanostructure transition. The effects of varying surfactants (cetyltrimethylammonium bromide (CTAB), and sodium dodecyl sulfate (SDS)), and the selective swelling of P2VP block via ethanol are also studied in detail, interested in discovering the morphology landscape of halogen‐bond regulated polymer assembly within the confined droplets. Computer simulations related to the experiments are also carried out to model and interpret the obtained morphologies, highlighting the molecular principles that govern the self‐assembly and structural transformation under 3D confinement.

## 2 Results and Discussion

### 2.1 Morphologies Generated from the Control Group

**Figure 2:**
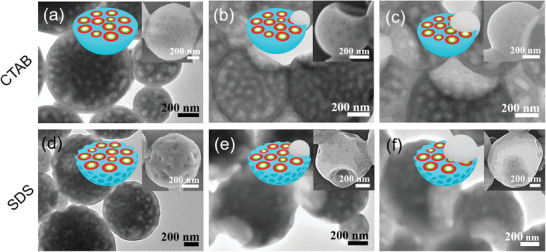
TEM images of PS‐*b*‐P2VP‐*b*‐PEO blended with different molar ratios of PPFPA using CTAB (top) or SDS (bottom) as an emulsifier, allowing the evaporation of CHCl_3_ at 30 °C for 24 h. *x* = 0 (a,d); *x* = 0.5 (b,e); *x* = 1 (c,f). PS matrix appears grey due to the high own contrast of PS in TEM, the PPFPA cores appear bright, and the P2VP cycles are dark due to I_2_ staining. Insets in the upper‐right are the cartoons showing the nanostructured particles, sky blue, red, green, white, and gray colors represent PS, P2VP, PEO, cavities, and macrophase‐separated PPFPA domain, respectively.

Prior to the study the halogen‐bond driven confined assembly between PS‐*b*‐P2VP‐*b*‐PEO and PTFIPA, topologies generated from the mixture of PS‐*b*‐P2VP‐*b*‐PEO with a control polymer PPFPA (poly(3‐(perfluorophenoxy)propylacrylate)) is analyzed. As a control polymer, PPFPA bears perfluorophenoxy in place of iodotetrafluorophenoxy group, thus devoid of specific halogen‐bonding interaction. Amphiphilic triblock copolymer PS‐*b*‐P2VP‐*b*‐PEO and varied amounts of PPFPA homopolymer are primarily mixed in chloroform, which is then emulsified with surfactant (CTAB or SDS) aqueous solution. The subsequent solvent evaporation and removal can induce the instabilities of the chloroform/water interface, thus facilitating the generation of microparticles with unique hierarchical structures (**Figures** **2** and S1, Supporting Information).

Pristine PS‐*b*‐P2VP‐*b*‐PEO shows internal porous spherical particles, consisting of intensive cavities (*d* = 60 ± 9 nm) when CTAB is employed as a surfactant (Figure 2a). The generation of such scattered cavities reveals that water droplets are formed inside the amphiphilic PS‐*b*‐P2VP‐*b*‐PEO triblock copolymer particles. These porous structures' formation can be attributed to water diffused into particles, dissolving the PEO block and thus forming droplets because of the relatively low volume fraction of the PEO block (*f*_PEO _= 0.13). The dark cycles surrounding these pores are the signature of P2VP microdomains, due to the selective staining of P2VP block by I_2_ vapor. The frame of porous particles is ascribed to the PS segment, demonstrating as the gray matrix of particles (Figure 2a). We then mix PS‐*b*‐P2VP‐*b*‐PEO with the control polymer PPFPA in specific PFPA/2VP molar ratios (*x* = 0.5 and 1, *x* represents the molar ratio of PFPA to 2VP units). Upon blending of a small amount PPFPA (*x* = 0.5) with PS‐*b*‐P2VP‐*b*‐PEO, notched sphere‐like structures are formed (Figure 2b). Increasing PPFPA content (*x* = 1) results in the expansion of the PPFPA domain, therefore larger notches are generated from these particles (Figure 2c). These results can be ascribed to the repulsion forces between PPFPA and PS‐*b*‐P2VP‐*b*‐PEO. Moreover, the comparable affinity of CTAB toward hydrophobic PS and PPFPA prevents PPFPA that can be fully encapsulated via PS‐*b*‐P2VP‐*b*‐PEO during solvent evaporation. Moreover, the formation of notched sphere‐like structures implies that PPFPA can be removed together with the surfactant during the purification and centrifugation process.

In contrast, the surface morphology of internal porous particles from pristine PS‐*b*‐P2VP‐*b*‐PEO emulsified with SDS is more irregular (Figure 2d), the collapsed surface could be the evidence of interfacial instability. Adding a small amount of PPFPA to PS‐*b*‐P2VP‐*b*‐PEO, internal porous particles again convert to a notched sphere‐like structure (Figure 2e). Increasing the content of PPFPA also results in the expansion of the PPFPA domain (Figure 2f).

### 2.2 Effects of Halogen‐Bond Interaction on Particles Morphology

**Figure 3:**
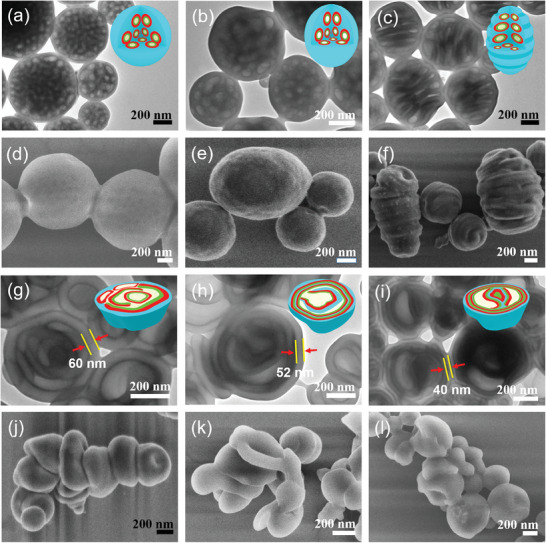
TEM (a–c,g–i) and SEM (d–f,j–l) images of PS‐*b*‐P2VP‐*b*‐PEO blended with different molar ratios of PTFIPA using CTAB as a surfactant, allowing the evaporation of CHCl_3_ at 30 °C. *x* = 0, large‐size particles with scattered cavities (a,d); *x* = 0.25, ellipsoidal microparticles with scattered cavities (b,e); *x* = 0.5, ellipsoidal particles with internal multi‐chamber and corrugated surface topologies (c,f); *x* = 0.75, particles internal multi‐chamber architectures (g,j); *x* = 1, particles internal multi‐shell architectures (h,k); *x* = 2, concentric particles with layer separation (i,l). Insets in the upper‐right are the cartoons showing the nanostructured particles, sky blue, red, green, and white color represent PS, halogen‐bonded P2VP/PTFIPA domain, PEO, and cavities, respectively.

Having established the macrophase separation from the blend of PS‐*b*‐P2VP‐*b*‐PEO with the control polymer PPFPA, we turn the focus to halogen‐bond mediated 3D confined‐assembly between PS‐*b*‐P2VP‐*b*‐PEO and PTFIPA (**Figures** **3** and S2, Supporting Information). Indeed, compared to the internal porous spherical particles from pristine PS‐*b*‐P2VP‐*b*‐PEO (Figure 3a,d), we notice a slightly increased internal dark layer, along with the transition of particles from spherical (Figure 3a,d) to ellipsoidal particle configuration (Figure 3b,e), when trace amount of PTFIPA is added (TFIPA/2VP molar ratio *x* = 0.25). The internal cavities of particles are again due to the volatilization of droplets from PEO/H_2_O. The outermost grey layer and frame are corresponding to the PS matrix, while dark walls between the PS domain and cavities are formed by halogen‐bonded P2VP and PTFIPA.

Further increasing PTFIPA content (TFIPA/2VP molar ratio *x* = 0.5) can enlarge the P2VP domain, thereby induce the increase of packing parameter. Consequently, the morphological conversion is initiated until an equilibrium state is established, indeed, ellipsoidal particles with internal multi‐chamber topologies are dominantly formed. Such internal multi‐chamber architectures are resulted from the enlarged and coalesced internal water droplets, which are arranged along the direction of the long axis (Figure 3c). Water droplets can evaporate via diffusion through the particles surface, leading to the generation of internal cavities. As the water evaporation process goes on, the particles prone to shrinkage and their skin buckles to form corrugated surface topologies (Figure 3f). To the best of our knowledge, these structurally novel ellipsoidal particles with internal multi‐chamber and corrugated surface topologies described above are the latest additions to the family of BCP morphology.

When x continues to increase to 0.75, further increased packing parameter leads to the internal cavities of particles continuing expands and merges into larger multi‐chamber structures (Figure 3g). If the TFIPA/2VP ratio is increased to 1, increased packing parameter that facilitates the formation of multi‐shell particles (Figure 3h). It is noteworthy that the internal multi‐chambers are too big to maintain the frame of particles, accordingly, collapsed particles, such as red‐blood‐cell‐like plates are observed from SEM investigations (Figure 3j,k).

In the case of TFIPA/2VP ratio is increased to 2, the number of spherical layers increases dramatically, leading to the formation of concentric particles (Figure 3i). Moreover, the increased P2VP domain along with the unchanged PS volume, lead to a loose arrangement of the PS chain; as a result, the grey PS domain became thinner than that as shown in Figure 3g,h. The addition of an excess amount of PTFIPA relative to P2VP blocks does not lead to macrophase separation, as excess PTFIPA chain can be dissolved into the PTFIPA/P2VP mixed phase, which explains the fact that PS‐*b*‐P2VP‐*b*‐PEO/PTFIPA blends demonstrate uniform microphase separated structures covering a wide composition range (*x* = 0 → *x* = 2).

It is known that the packing parameter or volume fraction that determines the phase segregation structure which is closely correlated to the block ratio of copolymers. Thanks to the halogen‐bond driven supramolecular association within the emulsion droplets, gradually increasing the adding amount of PTFIPA to PS‐*b*‐P2VP‐*b*‐PEO can systematically increase the volume fraction of the P2VP block while devoid of the tedious synthesis workload. Therefore, the facile engineering of packing parameters and control of the morphology evolution sequence of hollow particles is indeed achieved via strategically supramolecular design. Such morphology evolution of multilevel hollow‐structured particles, from porous, multi‐chamber, multi‐shell, to concentric multi‐layer architecture, are induced by the gradual increasing of polymer packing parameters, which is undoubtedly dominated by halogen‐bonding interactions between PS‐*b*‐P2VP‐*b*‐PEO and PTFIPA during 3D confined self‐assembly.

**Figure 4:**
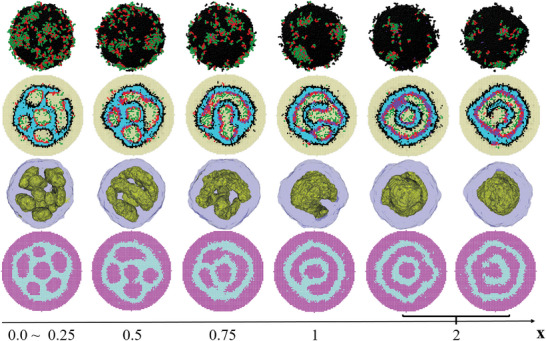
Self‐assembled morphologies obtained from simulations for the model CTAB(E_2_F_1_) system as a function of the molar ratio of D(TFIPA) to B(2VP) monomers, *x*. For each particle, a close‐up view and a cross‐section view of all components, as well as an isosurface contour view and a combining cross‐section view are shown. The two cross‐section views are shown in two perpendicular directions and the degenerate morphologies are shown in the brace. Color scheme in the close‐up and cross‐section views of all components: A (sky blue), B (red), C (green), D (purple), E and F (black), water (yellow); in the isosurface contour view: water inside the particle (yellow), and all the other chemical species (slate blue); in the combining cross‐section view: A and E_2_F_1_ (light blue) and all the other chemical species (violet).

Self‐assembled morphologies obtained from simulations for the model CTAB(E_2_F_1_) system are shown as a function of the molar ratio of D(TFIPA) to B(2VP) monomers, x in **Figure** **4**, where the concentration (*φ*) of E_2_F_1_ is fixed at 0.11. Notably, the morphology of each particle is composed of several water‐domains dispersed in the continuous A‐domain, which is due to the solvent evaporation‐induced diffusion of water into the inner of the particles, such result is also agreed with the experimental ones (Figure 3). To reduce the contact between A and water, E_2_F_1_ molecules and B monomers are usually at the A/water interfaces and C‐blocks are mainly dissolved in the water‐domains. In particles with homopolymers D_4_, that is, *x* > 0, due to the attractive interaction (formation of halogen‐bonding interaction) between them, D and B monomers tend to close to each other. On the other hand, due to the hydrophobic nature of D monomers, B monomers are usually at D/water interfaces. When *x* ≤ 0.5, D monomers usually spread into a mono‐layer between A and B domains, while when *x* > 0.5, small domains composed of only D and B monomers are observed inside the water‐domain. Specifically, in particles without or with a little amount of homopolymer D_4_, that is, *x *≤ 0.25, several nearly spherical and a few elongated water‐domains surround a nearly spherical central water‐domain, similarly to the internal porous particles observed in experiments (Figure 3a,b). Different water‐domains are interconnected through narrow water‐channels. As x increases, the water‐domains coalesce, forming internal multi‐chamber architectures determined in experiments (Figure 3c,g). When *x* = 0.5, besides the nearly spherical central water‐domain, curved rod‐like water‐domains are observed. When *x* = 0.75, a water‐domain is just like a small curved‐sheet and from its edge some rod‐like water‐domains are emitting. At *x* = 1, the outermost water‐domain inside the particle constitutes a spherical shell but with some irregular holes, where the holes are filled with A‐domains which span the inner and the outer A‐domains making them together as a continuous morphology, similarly to multi‐shell particles observed in experiments (Figure 3h). At *x *= 2, roughly concentric A‐water lamellar morphology is observed where the outer water‐domain constitutes a spherical shell but with some irregular holes filled with domains composed of B/D. The major difference between the degenerate morphologies at *x* = 2 is whether there are holes (filled with water‐domains) in the inner A‐layer. Such morphologies obtained from simulations (Figure 4) are qualitatively consistent with those observed in experiments (Figure 3), which can explain the step‐wise morphology conversion of multilevel hollow‐structured particles. The particle shapes (*x *= 0.25, 0.5), however, are spherical rather than ellipsoidal observed in the experiment, which may be due to the relatively smaller size of the simulation system compared to the experimental one.

Figure 4 also shows that with the increase of *x*, the morphology of water‐domains shifts to that with smaller A/water (or D/water) interfaces; that is, the number of water‐domains inside each particle decreases while their average size increases. The variation trend of the morphology of water‐domains with *x* in Figure 4 is mainly due to the attraction between B and D, which drives some B monomers away from the A/water (or D/water) interfaces. To reduce the contact between A (or D) and water, the morphology of water‐domains shifts to that with smaller A/water (or D/water) interfaces. The variation of the average contact number between B and water, with *x* can prove this result (Figure S5, Supporting Information).

In the outermost A/water interface of each particle, E_2_F_1_ molecules spread into a thin layer where embedded some discrete B‐blocks and a few B‐patches to separate A/water contact, while C‐blocks connected with those B‐blocks are dissolved in the outside water. Furthermore, it is noted that the amount of the outside C‐blocks or B‐blocks decreases with the increase of *x*, which is also due to the attraction between B and D. On the other hand, in the inner‐side of the outermost A‐domain, B‐blocks usually spread into a thin layer on the A/water (or D/water) interface, where embedded some E_2_F_1_ molecules. Furthermore, for the inner A‐domain, the case of the A/water or D/water interface is similar to that in the outermost A‐domain, that is, the outside A/water or D/water interface is mainly composed of E_2_F_1_ molecules while its inner‐side A/water or D/water interface is mainly composed of B‐blocks. This trend becomes increasingly pronounced with the increase of *x* (Figure 4). These features are consistent with the experimental results.

The size distribution of microparticles of PS‐*b*‐P2VP‐*b*‐PEO blended with diﬀerent molar ratios of PTFIPA (x) using CTAB as emulsifier are shown in Figure S3 (Supporting Information). In all cases, large‐size particles (400–800 nm) were observed, as well as their size distribution in the 10–20% range.

### 2.3 Effects of Swelling/Deswelling on Particles Morphology

**Figure 5:**
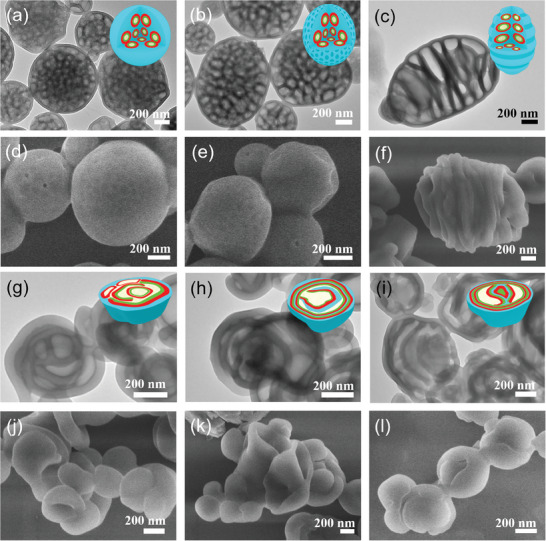
TEM (a–c,g–i) and SEM (d–f,j–l) images of PS‐*b*‐P2VP‐*b*‐PEO blended with different molar ratios of PTFIPA using CTAB as a surfactant, which are dispersed in ethanol at 30 °C for 24 h: *x* = 0, sphere particles with larger‐sized cavities (a,d); *x *= 0.25, sphere particles with larger‐sized cavities (b,e); *x* = 0.5, particles with enlarged internal multi‐chamber cavities (c,f); *x* = 0.75, particles with enlarged internal multi‐chamber cavities (g,j); *x* = 1, multi‐compartment vesicles with twisted shell topologies (c,g); *x* = 2, multi‐compartment vesicles with twisted shell topologies (d,h). Insets in the upper‐right are the cartoons showing the nanostructured particles, sky blue, red, green, and white colors represent PS, halogen‐bonded P2VP/PTFIPA domain, PEO, and cavities, respectively.

To assess the merits of stimuli‐responsive particles in a selective solvent, such as ethanol, the swelling/deswelling process is subsequently applied to reengineer the multi‐level hollow particles. Indeed, when the porous particles generated from pristine PS‐*b*‐P2VP‐*b*‐PEO are treated by ethanol, a larger cavity structure can be obtained (**Figure** **5**a). Ethanol is a good solvent for P2VP blocks, while poor solvent for PS and PTFIPA blocks. Therefore, ethanol may result in selective swelling of the P2VP block. Moreover, due to the emerged pressure from the expanded P2VP domain, non‐expended PS and PTFIPA blocks matrix are therefore extruded to induce the plastic deformation. Polymer particles on the copper mesh from TEM grids may be sunk after volatilization of ethanol, resulting in a volume increase of internal cavities comparing to original multi‐level hollow particles. Nevertheless, rigid PS and PTFIPA domains within particles can maintain the frame of reconstructed particles, due to their high glass transition temperature (*T_g_*) and poor solubility in ethanol. As a result, particles with larger‐sized cavities can be determined from PS‐*b*‐P2VP‐*b*‐PEO after swelling/deswelling of ethanol (*x* = 0, 0.25, Figure 5a,b). Increasing P2VP/PTFIPA molar ratio (*x* = 0.5, 0.75) leads to further extruded and expanded internal cavities, accordingly, enlarged internal multi‐chamber cavities (Figure 5c,g) with intensively corrugated surfaces (Figure 5f,j) are determined. If *x* is further increased to 1 and 2, swelling of the P2VP layer by ethanol results in volume increase, subsequent ethanol volatilization leads to the expansion of layers, thus forming multi‐compartment vesicles with twisted shell topologies (Figure 5h,i).

### 2.4 Effects of Surfactants on Particles Morphology

**Figure 6:**
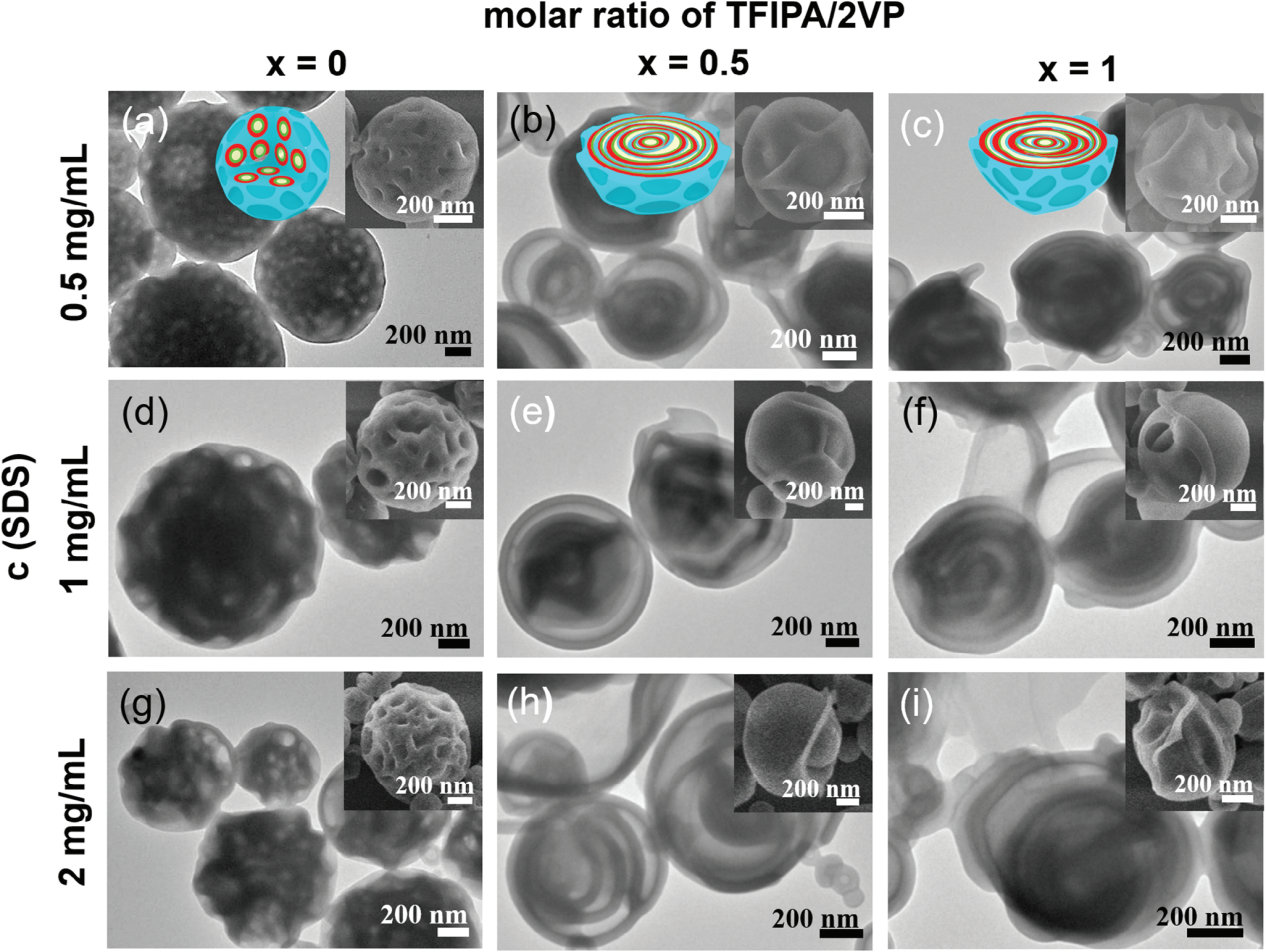
TEM and SEM (inset) images of PS‐*b*‐P2VP‐*b*‐PEO blended with different molar ratios of PTFIPA (*x*) using various concentrations (c) of SDS as surfactant, allowing the evaporation of CHCl_3_ at 30 °C. *c *= 0.5 mg mL^−1^, *x* = 0 (a); *c* = 0.5 mg mL^−1^, *x* = 0.5 (b); *c *= 0.5 mg mL^−1^, *x* = 1 (c); *c* = 1 mg mL^−1^, *x* = 0 (d); *c* = 1 mg mL^−1^, *x* = 0.5 (e); *c* = 1 mg mL^−1^, *x* = 1 (f); *c* = 2 mg mL^−1^, *x *= 0 (g); *c* = 2 mg mL^−1^, *x* = 0.5 (h); *c* = 2 mg mL^−1^, *x* = 1 (i). Insets in the upper‐right are the cartoons showing the nanostructured particles, sky blue, red, green, and white colors represent PS, halogen‐bonded P2VP/PTFIPA domain, PEO, and cavities, respectively.

Compared to cationic surfactant (e.g., CTAB), synergistic adsorption of PS‐*b*‐P2VP‐*b*‐PEO BCPs and sodium dodecyl sulfate (SDS) to the surface of the emulsion droplet induced a dramatic decrease in the interfacial tension and generated interfacial instability at the particle surface may lead to undulant geometrical shape at the interface.^[^ ^37^ ^]^ Therefore, the morphological evolution of particles from halogen‐bonded PS‐*b*‐P2VP‐*b*‐PEO/PTFIPA system, using SDS as surfactant by increasing of TFIPA/2VP (x) and concentration (c) of SDS is subsequently studied (**Figures** **6** and S4, Supporting Information). Confined‐assembly of pristine PS‐*b*‐P2VP‐*b*‐PEO at SDS concentration (c) of 0.5 mg mL^−1^ affords particles with internal intensive spherical cavities (Figure 6a), along with the highly corrugated surfaces revealed by SEM studies (Figure 6a, inset). Adding PTFIPA to PS‐*b*‐P2VP‐*b*‐PEO (*x* = 0.5) lead to the increased volume fraction at the P2VP block. Accordingly, concentric particles with water‐filled cavities are observed, showing the mixed cylindrical and layered structures (Figure 6b). The dehydration and shrinkage of the PEO/H_2_O domain again afford the winkle surfaces (Figure 6b, inset). When *x* = 1, further increased packing parameters facilitate the transformation toward multilayered structures of such halogen‐bonded particles (Figure 6c).

In addition, the increasing of SDS concentration will lead to the promoted emulsification capability, thus may impact the change of chain arrangement and particle structure. If SDS concentration is increased from 0.5 to 1 mg mL^−1^, promoted emulsification will enable more water diffused into particles, accordingly, the interior cavities of particles are expanded (Figure 6d), along with the growing wrinkles (Figure 6d, inset) compared to Figure 6a. Intensive wrinkles further deepen with increasing SDS concentration to 2 mg mL^−1^, illustrating more irregular particle morphology (Figure 6g).

Upon adding PTFIPA to PS‐*b*‐P2VP‐*b*‐PEO, spherical particles with internal cavities transformed into multi‐compartment vesicle architectures, displaying disordered hydrated lamellar layers or cylindrical channels (Figure 6e,f,h,i). The formation of multi‐compartment vesicles can be understood because of the increased polymer packing parameter and promoted emulsification capability. It is noteworthy that in the case of *x* = 1, *c*(SDS) = 2 mg mL^−1^, the lamellar layers of multi‐compartment vesicles are intensively dispersed, even disassembled (Figure 6i), along with the twisted and deformed surface morphology (Figure 6i, inset).

**Figure 7:**
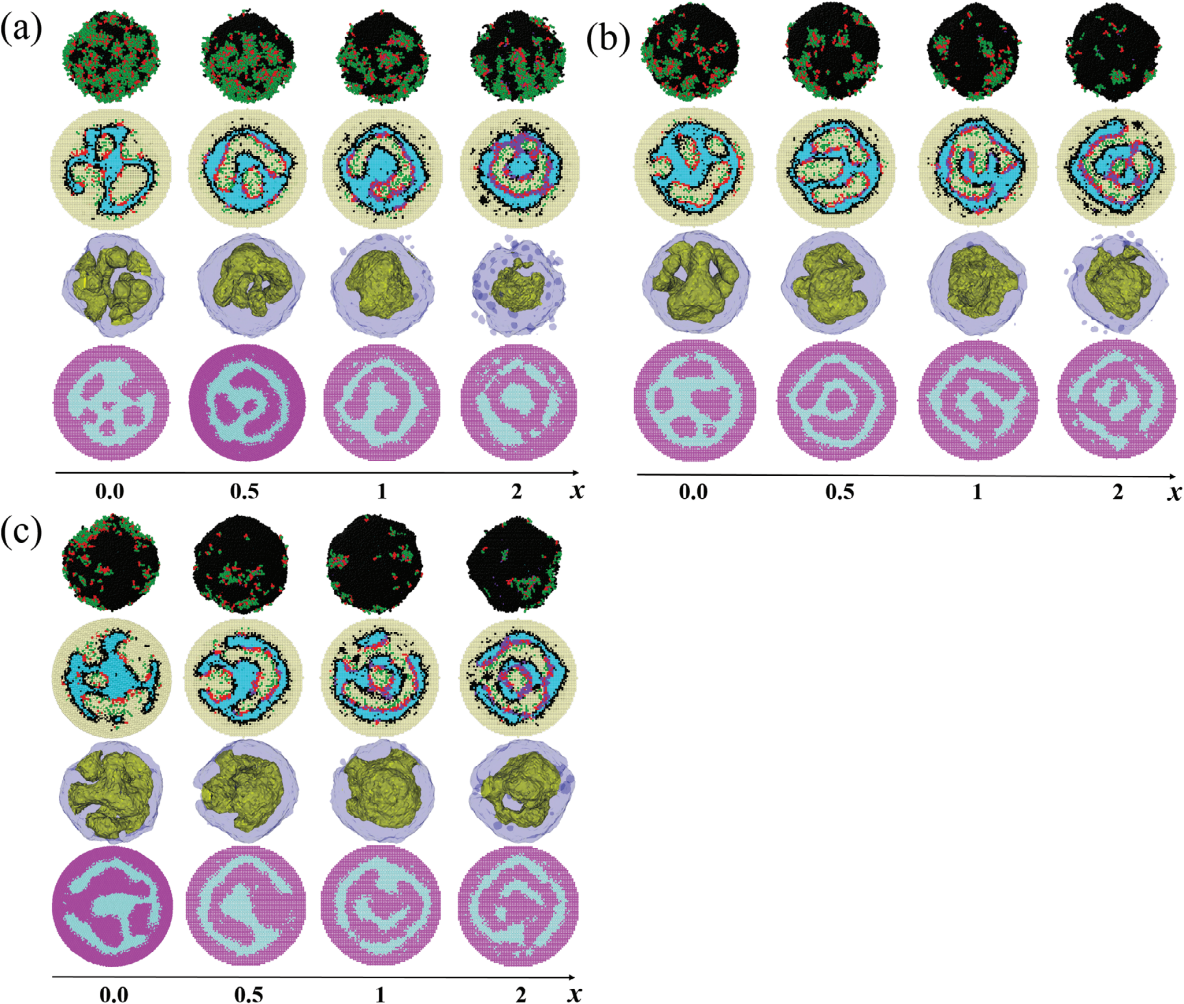
Self‐assembled morphologies obtained from simulations for the model SDS systems as a function of *x* at different SDS concentration *φ*. a) *φ* = 0.09, b) *φ* = 0.11, c) *φ* = 0.13. The color schemes are the same as those in Figure 4.

Self‐assembled morphologies obtained from simulations for the model SDS(E_2_F_1_) systems as a function of x are shown in **Figure** **7** for three SDS concentrations. Compared with that of CTAB, the head of SDS exhibits enhanced selectivity toward C‐block and is more soluble in water, hence the interaction between C and F, and that between F and W are stronger in systems with SDS (*ɛ*_CF _= −2.5 and *ɛ*_FW _= −1.5). Because of the stronger hydrophilicity of F, a small portion of E_2_F_1_ molecules in SDS systems form small micelles inside the water domain outside the particle, besides at the A/water (or D/water) interfaces when *x* = 1,2, as shown in the cross‐section view in Figure 7. The same as that in the CTAB system, C‐blocks are dissolved in the water‐domains, and with the increase of *x*, the morphology of water‐domains shifts to that with smaller A/water (or D/water) interfaces. Furthermore, at a given *x*, the morphology of water‐domains also shifts to that with smaller A/water (or D/water) interfaces compared to that of the CTAB system. Specifically, in the model SDS system with *φ* = 0.09, several nearly spherical and a few elongated water‐domains are observed in the particle at *x* = 0, similarly to the morphology observed in the CTAB system at *x* = 0 and experiments (Figure 6a). At *x* = 0.5, the particles are composed of a small curved‐sheet‐shaped water‐domain connected with some rod‐like water‐domains which is similar to the morphology obtained in the CTAB system at *x *= 0.75 and the experimental one (Figure 6b). At *x* = 1 and 2, continuous morphology and roughly concentric A‐water lamellar morphology are observed, respectively, which are similar to those obtained in the CTAB system at the corresponding *x* values. When *x* = 1, the morphology is in an intermediate state of transformation toward multilayered structures of such halogen‐bonded particles, consistent with the experimental observations (Figure 6c). When *φ* = 0.11 and 0.13, only continuous morphology and roughly concentric A‐water lamellar morphology are observed. It is interesting to note that the outermost water‐domain (inside the particle) in the continuous morphology obtained at *x *= 0 in the case of *φ *= 0.11 and 0.13 is composed of triple junctions (Figure 7b,c), that is, having the characteristic of gyroid structure. Again, the simulation results (Figure 7) are qualitatively consistent with the experimental ones (Figure 6).

**Figure 8:**
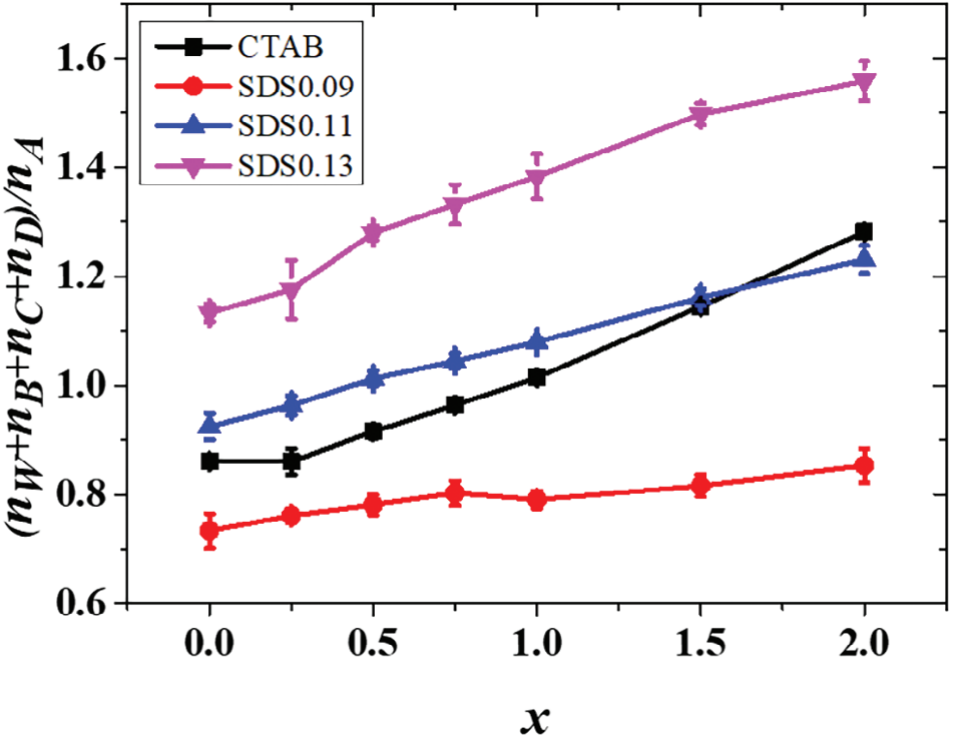
The x dependence of the ratio of the effective volume fraction of water domains to the volume fraction of A‐blocks inside a particle, where *n*_W_, *n*_A_, *n*_B_, *n*_C,_ and *n*_D_ are the number of water molecules, A‐monomers, B‐monomers, C‐monomers, and D‐monomers, respectively, inside a particle.

The *x* dependence of the ratio of the effective volume fraction of water domains to the volume fraction of A‐blocks inside a particle is shown in **Figure** **8**. As both C‐blocks and the domains composed of D and B monomers are all inside the water domains of a particle, hence they all contribute to the effective volume fraction of water domains. It is noted in Figure 8 that the relative effective volume fraction of water domains inside a particle increases with the increase of the surfactant concentration in the system. This relative effective volume fraction is often slight higher in an SDS system than that in the CTAB system with the same surfactant concentrations, which is due to the stronger attraction between F‐ and W‐monomers in the SDS system, resulting in the relatively easier diffusing of water into the particle. In the CTAB system, the variation trend of morphology of the water‐domain with *x*, from nearly spherical to rod‐like to continuous to concentric morphologies (Figures 3, 4), is similar to that in the spherically‐confined diblock copolymer systems with the increase of volume fraction of one block. Therefore, it is deduced that the increase of the relative effective volume fraction of water‐domains with *x* is the reason of the morphology variations in Figure 3 and Figure 4. This conclusion is also true for the SDS system. A morphological sequence similar to that observed in the CTAB system is obtained in the system with lower SDS concentration (Figure 6a–c and Figure 7a) for the same reason. Whereas for systems with higher SDS concentrations, their higher relative effective volume fraction of water‐domain (even at *x* = 0) results in the lack of spherical and rod‐like morphologies (Figure 6d–i and Figure 7b,c). On the other hand, for a given morphology, the window of the effective volume fraction of the water‐domain may not be the same as that in the diblock copolymer system. This is because the morphologies are obtained from solvent evaporation in both our experiments and our simulations, instead of in equilibrium.

## 3 Conclusion

Based on the studies combining experiments and simulations, halogen‐bond driven 3D confined‐assembly toward multi‐level hollow particles with tunable shape and internal structure are systematically engineered, by tuning the PTFIPA content, on the choice of surfactant, and P2VP swelling/deswelling process. Certain morphologies obtained in current work have not been discovered in the literature yet. Polymer packing parameters and Monte Carlo simulations provide a reliable way to understand the dependence of morphology transition sequence. We conclude that the transition of multilevel hollow‐structured particles and halogen‐bonding interactions are closely related, demonstrating that using halogen‐bond in 3D confined polymer self‐assembly is a vibrant new field of research. These multilevel hollow particles serve to demonstrate the significance and potential that halogen‐bond can contribute in this field, which provide engaging opportunities where the internal structure has critical roles in determining their functions, in the context of, for example, encapsulation, nanoreactor, and catalyst support.

## Conflict of Interest

The authors declare no conflict of interest.

## Supporting information

Supporting Information

## Data Availability

The data that support the findings of this study are available from the corresponding author upon reasonable request.
