# Supplementary material for: Multilevel Hollow‐Structured Particles through Halogen‐Bond Regulated Polymer Assembly under 3D Confinement
Source: Adv Sci (Weinh). 2024 Sep 4;11(41):2405103. doi: 10.1002/advs.202405103 (PMC11538654; doi:10.1002/advs.202405103)
Supplement: Supplementary file 1 — Supporting Information [file ADVS-11-2405103-s001.docx]

Supporting Information for:

**Multilevel Hollow-Structured Particles** [**through Halogen-Bond Regulated Polymer Assembly under 3D Confinement**](https://pubs.acs.org/doi/abs/10.1021/acs.macromol.5b00335)**: Experiment and Simulation**

*Xihuang Zheng,^a^*^†^ *Yi Zhao, ^b^*^†^ *Yuping Zhang, ^a^ Renhua Deng,^a^ Baohui Li,^b^* Senbin Chen,^a^* and Jintao Zhu ^a^**

^a:^ School of Chemistry and Chemical Engineering, Huazhong University of Science and Technology (HUST), Wuhan 430074, China

^b^: Key Laboratory of Weak-Light Nonlinear Photonics, Ministry of Education, School of Physics, Nankai University, Tianjin 300071, China

^†:^ X. Z. and Y. Z. contributed equally to the work.

Correspondence to: J. Zhu (jtzhu@hust.edu.cn), S. Chen ([senbin@hust.edu.cn](mailto:senbin@hust.edu.cn)), B. Li ([baohui@nankai.edu.cn](mailto:baohui@nankai.edu.cn))

Table of Content

[Materials 3](#_Toc169984839)

[Preparation of the halogen bonded supramolecular polymer microparticles 3](#_Toc169984840)

[Preparation of the dissembled microparticles 4](#_Toc169984841)

[Characterization methods 4](#_Toc169984842)

[Simulation details 4](#_Toc169984843)

Materials

Triblock copolymer PS-*b*-P2VP-*b*-PEO (PS_75k_-*b*-P2VP_21k_-*b*-PEO_16.5k_, *Mn* = 112.5 kg/mol, PDI = 1.12) was purchased from Polymer Source, Inc. PTFIPA (*Mn* = 32.2 kg/mol) and PPFPA (*Mn* = 23.1 kg/mol) were synthesized in our previous report.^1^ Sodium dodecyl sulfate (SDS, 98%), cetyltrimethyl ammonium bromide (CTAB, 99%) were purchased from Aldrich. Other chemicals were purchased from Aladdin. All of the chemicals were used without further purification unless otherwise indicated.

# Preparation of the halogen bonded supramolecular polymer microparticles

Halogen bonded supramolecular polymer microparticles were prepared by using the emulsification method, followed by the solvent evaporation at 30 ^o^C. The PS-*b*-P2VP-*b*-PEO (PTFIPA)_x_ supramolecules were prepared by dissolving PS-*b*-P2VP-*b*-PEO and PTFIPA in chloroform, respectively (10 mg PS-*b*-P2VP-*b*-PEO in 1mL chloroform; 7.2 mg PTFIPA in 1mL chloroform). Then, the solutions were mixed together with desired volume ratio of 2VP/TFIPA (100 μL/0 μL; 80 μL/20 μL; 67 μL/33 μL; 57 μL/43 μL; 50 μL/50 μL; 33 μL/67 μL). Then, 0.1 mL of the mixed solution was emulsified with 1.0 mL aqueous solution of CTAB or SDS through a hand-driven membrane-extrusion emulsification device. The emulsion was collected in a 10 mL open vial to allow the slow evaporation of chloroform for 24 h at 30 °C, eventually leading to the solidification of polymers and segregation of the building blocks within the confined spaces. The particles were thus obtained through centrifugation of the resulting suspension to eliminate the redundant CTAB or SDS, and finally the obtained particles were dispersed in distilled water and subjected to TEM investigations.

PS-*b*-P2VP-*b*-PEO/PPFPA were prepared by the same methods, the concentration of PPFPA in chloroform is 5.3 mg/1 mL.

# Preparation of the dissembled microparticles

The fresh obtained microparticles were dispersed in excess ethanol and sonicated at 30 ^o^C for 1 h to selectively swell the P2VP domain. The centrifugation (14000 rpm, 10 min) is then applied to remove ethanol and the dissolved P2VP, the resulting objects were washed via ethanol (2 times), and re-dispersed in distilled water, leading to the mesoporous microparticles and nano-objects, which were subsequently subjected to the microscope investigations.

# Characterization methods

Transmission electron microscopy (TEM) analyses were conducted with a FEI TecnaiG2 20 transmission electron microscope. The samples were dispersed in DI water and spread onto a Cu grid coated with a Carbon-film. After 1 min, excess solution was blotted off with filter paper and air-dried before the measurement.

Scanning electron microscope (SEM) were conducted with a Sirion 200 scanning electron microscope. Samples of polymer nanoparticles in DI water were spread onto a precleaned silicon wafer, followed by drying in air at room temperature.

# Simulation details

Simulations are carried out to elucidate the details of the internal structures of the particles. We use Monte Carlo based solvent evaporation method^2^ and the single-site bond fluctuation model. Our simulations are aimed at the experimental systems. The model and the method have been demonstrated to be efficient for studying the self-assembly of block copolymers under solvent evaporation in our previous studies, and their details can be found elsewhere.^2^ Specifically, the model system consists of five components: triblock copolymer, homopolymer, surfactant molecule, organic solvent molecule (S), and water molecule (W). PS-b-P2VP-b-PEO triblock copolymer and PTFIPA homopolymer are therefore modelled as A_17_B_4_C_3_ and D_4_, respectively, after weighed the volume of these different monomers. Surfactant (CTAB or SDS) is modelled as E_2_F_1_, where E and F respectively representing the hydrophobic tail and hydrophilic head. The systems are confined in a spherical pore of diameter D_0_. The pore is embedded in a simple cubic lattice of volume V = L^3^ with L = D_0_+3. The monomers on the polymer chains and solvent molecules can only occupy the lattice site inside the pore, while lattice sites outside the pore constitute the pore wall which cannot be occupied by any monomers or molecules. Each monomer, each S molecule or each W molecule can only occupy one lattice site, and any two of them cannot occupy the same site simultaneously. The bond length is set to be 1 and √2 lattice spacing, so that each site has 18 nearest neighbors. Only the 18 nearest-neighbor interactions are considered during our simulations. The interaction parameters between different chemical species in each simulation system are fixed as follows: ɛ_AB_ =ɛ_AD_ =ɛ_BC_ =ɛ_CD_ = 0.5, ɛ_AC_ = 1 and ɛ_BD_ = -0.6, which indicates that any two different species between A, B, C and D are incompatible except between B and D, the negative value of ɛ_BD_ is due to the halogen-bonding interaction between B- and D- monomers; ɛ_AS_= -1.5, ɛ_BS_ =ɛ_DS_ =ɛ_ES_ =-1, ɛ_CS_ =-0.5, ɛ_FS_ =1, ɛ_AW_ =6, ɛ_BW_=0, ɛ_CW_=-0.5, ɛ_DW_= ɛ_EW_=1, and ɛ_SW_=4; which indicates that the solvent (S) is good for all monomers except the head of the surfactant; ɛ_AE_=-0.5, ɛ_BE_= 3, ɛ_CE_ =7, ɛ_DE_ =0, ɛ_AF_= ɛ_DF_ = ɛ_EF_=1, ɛ_BF_=-0.2; whereas ɛ_CF_, ɛ_FW_ varies with the surfactant use in the system. For CTAB system, we set ɛ_CF_=-1.5, ɛ_FW_=-1, while for SDS system ɛ_CF_=-2.5 and ɛ_FW_=-1.5 since that the head of SDS is more soluble in water and exhibits enhanced selectivity towards C-block. In all cases, it is assumed that interaction between the same species is zero. All the interaction parameters are in the unit of k_B_T, where k_B_ is the Boltzmann constant and T is the simulated temperature.

The initial conformation in our simulations is generated as follows. A spherical droplet of diameter D_d_, located in the center of the pore, is constructed. Within the droplet, polymer chains (including triblock copolymer and homopolymer chains) are randomly dissolved in S- solvent with an initial monomer concentration of c_0_ = 33%. The lattice sites between the spherical droplet and pore wall are occupied by aqueous solution of surfactant in which surfactant molecules are randomly distributed at a given concentration φ. Solvent is evaporated with a fixed rate, that is, we evaporate a randomly selected S-molecule that is at the oil−water interface after performing 6 Monte Carlo steps (MCS). After nearly all the S-molecules have been evaporated, snapshots of morphologies of the system are sampled and various quantities are calculated by ensemble average. In our simulations, k_B_T=3, D_0_ =72 and D_d_ = 62. The initial surfactant concentration φ varies in different systems. For each set of parameters, at least 5 independent simulations are performed with each being of a different seed for the random number generator. The results indicate that the resulted morphologies have good reproducibility.


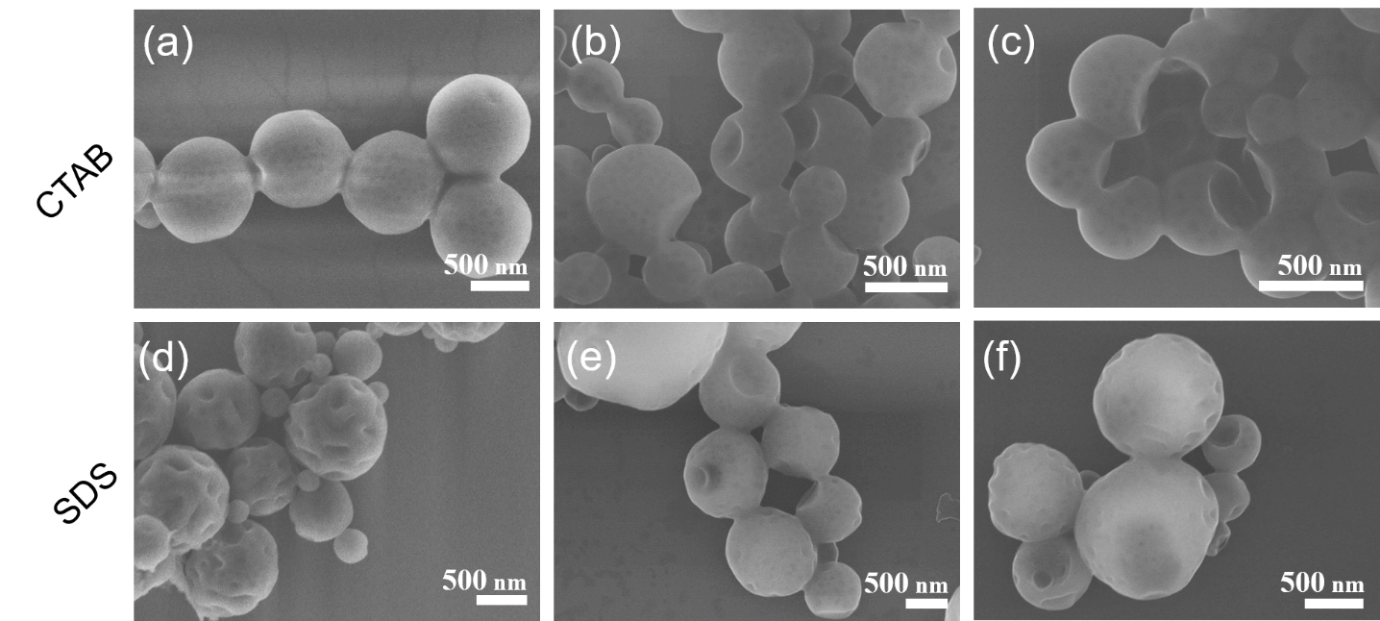


**Figure S1**: SEM images of PS-*b*-P2VP-*b*-PEO blended with diﬀerent molar ratios of PPFPA using CTAB (top) or SDS (bottom) as emulsifier, allowing the evaporation of CHCl_3_ at 30 ^o^C for 24 h. x = 0 (a&d); x = 0.5 (b&e); x = 1 (c&f).


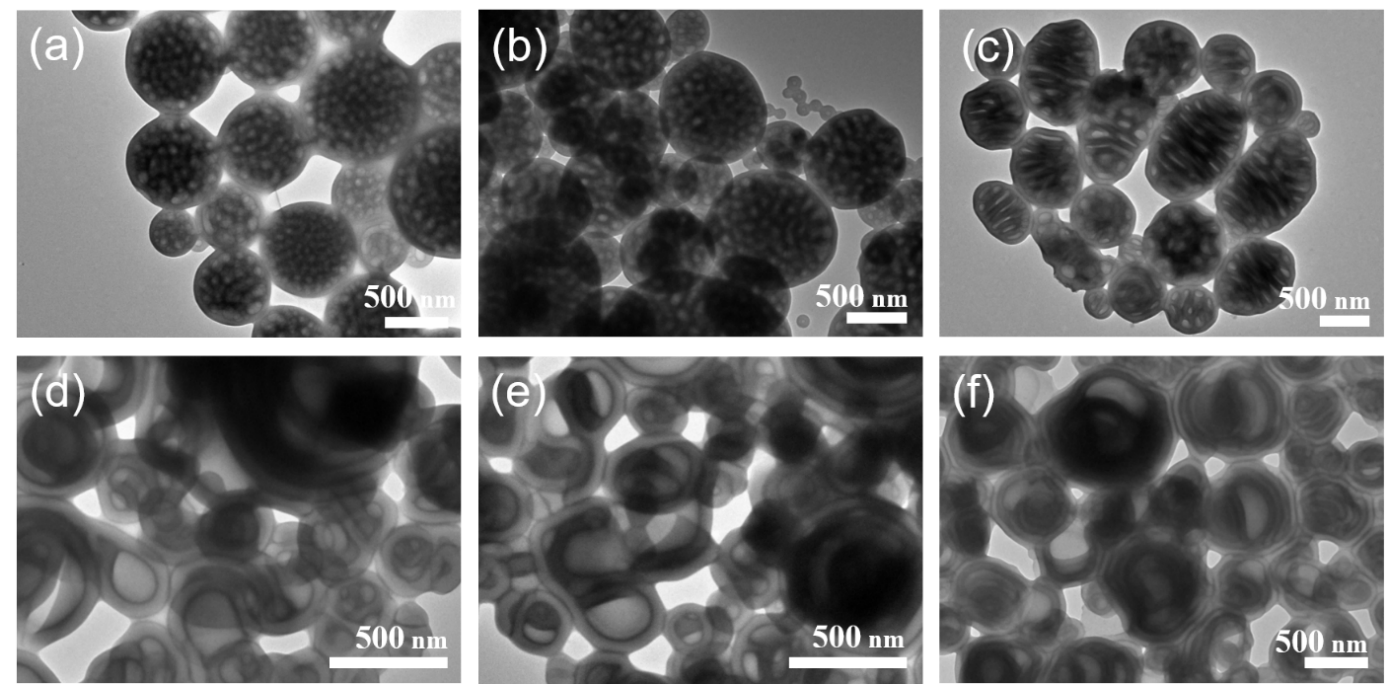


**Figure S2**: TEM images of PS-*b*-P2VP-*b*-PEO blended with diﬀerent molar ratios of PTFIPA using CTAB as surfactant at low magnification, allowing the evaporation of CHCl_3_ at 30 ^o^C. x = 0, large-size particles with scattered cavities (a); x = 0.25, ellipsoidal microparticles with scattered cavities (b); x = 0.5, ellipsoidal particles with internal multi-chamber and cor-rugated surface topologies (c); x = 0.75, particles internal multi-chamber architectures (d); x = 1, particles internal multi-shell architectures (e); x = 2, concentric particles with layer separation (f).


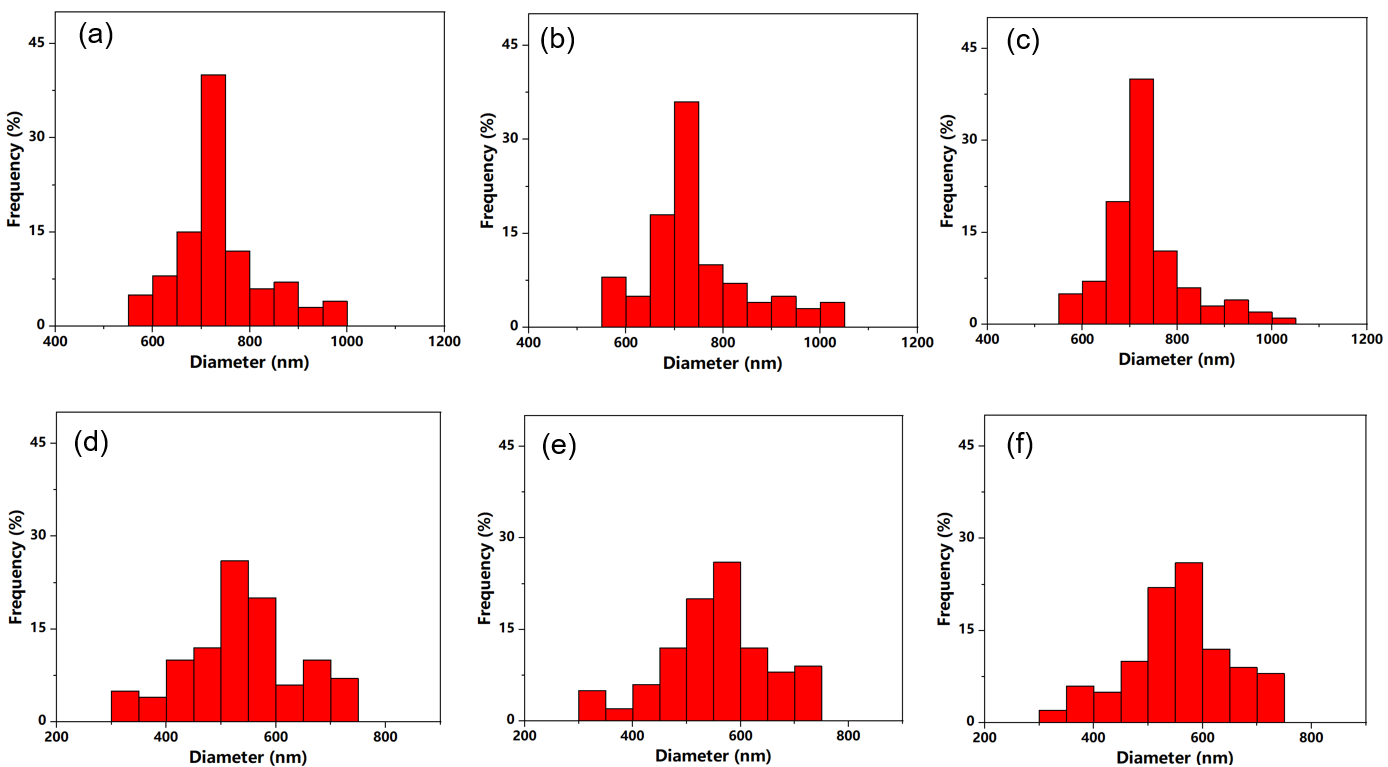


**Figure S3**: Size distribution of microparticles of PS-*b*-P2VP-*b*-PEO blended with diﬀerent molar ratios of PTFIPA (x) using CTAB as emulsifier. x = 0 (a); x = 0.25 (b); x = 0.5 (c); x = 0.75 (d); x = 1 (e); x = 2 (f).

.


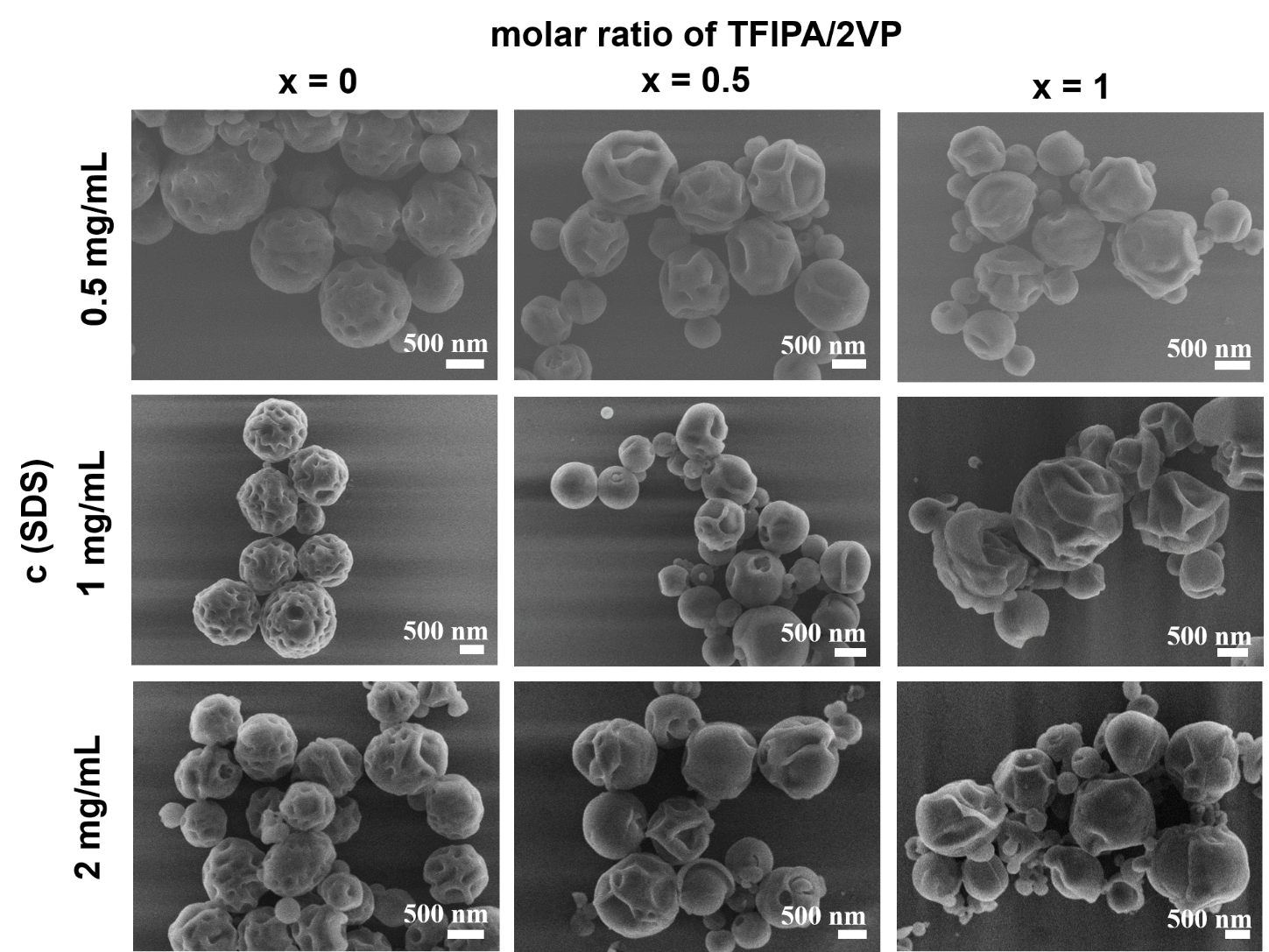


**Figure S4**: SEM images of PS-*b*-P2VP-*b*-PEO blended with diﬀerent molar ratios of PTFIPA (x) using various concentrations (c) of SDS as surfactant, allowing the evaporation of CHCl_3_ at 30 ^o^C. c = 0.5 mg/mL, x = 0 (a); c = 0.5 mg/mL, x = 0.5 (b); c = 0.5 mg/mL, x = 1 (c); c = 1 mg/mL, x = 0 (d); c = 1 mg/mL, x = 0.5 (e); c = 1 mg/mL, x = 1 (f); c = 2 mg/mL, x = 0 (g); c = 2 mg/mL, x = 0.5 (h); c = 2 mg/mL, x = 1 (i).

**Figure S5**: The average contact number *CN_BW_* as a function of x for the model systems.

**REFERENCES**

[1] X. Zheng, M. Ren, H. Wang, *et al.* Halogen-bond mediated 3d confined assembly of ab diblock copolymer and C homopolymer blends. *Small* **2021**, *17*, 2007570.

[2] L. Zheng, Z. Wang, Y. Yin, *et al.* Formation mechanisms of porous particles from self-assembly of amphiphilic diblock copolymers inside an oil-in-water emulsion droplet upon solvent evaporation. *Langmuir* **2019**, *35*, 5902-5910.
